# Correlation of TGF-β signaling pathway gene polymorphisms with unexplained recurrent spontaneous abortion

**DOI:** 10.1097/MD.0000000000035697

**Published:** 2023-10-27

**Authors:** Huiqin Xue, Jinsong Jiang, Jingbo Gao, Min Guo, Qiaoyin Tang, Xinyan Li, Hongyong Lu, Xiayu Sun, Jianrui Wu, Yuping Zhang

**Affiliations:** a Department of Cytogenetic Laboratory, Children’s Hospital of Shanxi, Women Health Center of Shanxi, Affiliated Hospital of Shanxi Medical University, Taiyuan, People’s Republic of China; b Department of Paediatric Medicine, Shanxi Medical University, Taiyuan, People’s Republic of China; c Department of Obstetrics and Gynecology, Children’s Hospital of Shanxi, Women Health Center of Shanxi, Affiliated Hospital of Shanxi Medical University, Taiyuan, People’s Republic of China.

**Keywords:** flight mass spectrometry, gene polymorphisms, high-throughput sequencing, TGF-β signaling pathway, unexplained recurrent spontaneous abortion

## Abstract

**Background:**

The association of key genes in the transforming growth factor-β (TGF-β) signaling pathway and their gene polymorphisms with unexplained recurrent spontaneous abortion (URSA) is unclear.

**Objective:**

To investigate the association of gene polymorphisms related to the TGF-β signaling pathway in URSA women.

**Methods:**

The study population consisted of 80 women with URSA and 90 normal control women, of which 10 women with URSA and 10 normal control women underwent high-throughput sequencing to select loci, and the remaining 70 women with URSA and 80 normal control women underwent flight mass spectrometry experiments to verify gene loci polymorphism. A total of 7 polymorphic loci in interleukin-6 (IL-6), TGF-β1, TNF-α, SMAD1, and TNFRSF4 genes were screened by high-throughput sequencing combined with a review of databases. An SNP flight mass spectrometer (Mass ARRAY detection system) was applied to detect the polymorphisms and their frequencies in 70 women with URSA and 80 normal control women at the 7 gene loci.

**Results:**

Among the 7 loci of IL-6, TGF-β1, TNF-α, SMAD1, and TNFRSF4 genes, 2 loci were found to have significantly different allele and genotype frequency distributions between the 70 URSA and 80 normal controls, one was the IL-6 gene -174G/C locus (rs1800795), the risk of disease was 2.636 and 3.231 times higher in individuals carrying the C allele and CC genotype than in those carrying the G allele and GG genotype, respectively; the other was the TGF-β1 gene -509T/C locus (rs1800469), and the risk of disease was 1.959 and 3.609 times higher in individuals carrying the T allele and TT genotype than in those carrying the C allele and CC genotype, respectively. The remaining 5 genetic loci have no statistically significant.

**Conclusion:**

IL-6 gene -174G/C locus (rs1800795) genotype CC and allele C may be the causative factor of URSA, TGF-β1 gene -509T/C locus (rs1800469) genotype TT and allele T may be the causative factor of URSA, and polymorphisms of the 2 loci may be associated with URSA.

## 1. Introduction

Recurrent spontaneous abortion (RSA), is defined as three or more consecutive pregnancy losses before the fetal weight of 500 g or lower.^[1]^ This criterion was changed internationally to two or more consecutive spontaneous abortions before 20 weeks of gestation.^[2]^ The incidence of RSA is 5% of all pregnancies in China and abroad.^[3]^ The risk factors for RSA are mainly chromosomal abnormalities, thrombosis, infection, immune dysfunction, endocrine disorders, and uterine abnormalities, but unexplained RSA (URSA) still accounts for about 50% of all RSA women.^[4]^ Therefore, it is important to study the pathogenesis of URSA to eventually cure or prevent RSA in advance.

The main feature of the TGF-β signaling pathway is its environmental dependence. Depending on the concentration and type of ligand, target tissue, and developmental stage, members of the TGF-β family in turn transmit a variety of different signals, and abnormal regulation of TGF-β signaling can lead to developmental defects and human diseases.^[5]^ Kohei Miyazono used a cell culture model system to demonstrate that the TGF-β signaling pathway is an important regulator of the apoptotic process and induces cellular autophagy.^[6]^ TGF-β and its related growth factors play an important role in adult tissue homeostasis and embryogenesis through the regulation of cell proliferation, differentiation, migration, and death.^[7]^ The process of pregnancy is complex and the TGF-β signaling pathway is involved in many reproductive processes, such as folliculogenesis,^[8]^ ovulation,^[9]^ embryo-fallopian tube interactions,^[10]^ embryonic development,^[11]^ formation of the reproductive tract,^[12]^ and endometrium decidualize.^[13]^ The TGF-β signaling pathway has an important role in pregnancy.^[14]^

Our previous study on the expression of TGF-β related pathway genes in URSA abortion embryo tissues and induced abortion tissues showed that the mRNA expression of interleukin-6 (IL-6), transforming growth factor-β (TGF-β1) and SMAD1 genes was more than 2 times different between URSA and normal embryo tissues. It is suggested that the mRNA expression of these genes in the TGF-β signaling pathway is significantly different between URSA and normal embryonic tissues.^[15]^ However, it is unclear whether the polymorphisms of TGF-β1, SMAD1, and IL-6 genes in the peripheral blood of pregnant women are associated with URSA, which is the question to be addressed in this study.

## 2. Material

### 2.1. Research subjects

The study population was 80 women with URSA and 90 healthy women with normal pregnancy history attending the outpatient clinic of Shanxi Children’s Hospital from 2019 to 2021, with an age range of 25 to 38 years, all of whom were from the Chinese Han population, from North China. The gestational weeks of the case group and the control group were matched at 6 to 20 weeks of pregnancy. Inclusion criteria for the URSA group: clinical diagnosis of URSA and history of 2 or more unexplained spontaneous abortions. Exclusion criteria: karyotype and embryonic chorion chromosome abnormalities of both spouses; cardiopulmonary diseases; digestive system diseases; liver and kidney insufficiency; malignant tumors; hematological system diseases; autoantibodies and other abnormal immune factors; reproductive tract malformations; teratogenic drugs taken during pregnancy; hepatitis B, syphilis and other infectious diseases. Inclusion criteria for the control group: healthy women with normal pregnancy history with at least 1 to 2 live births and no history of miscarriage. Exclusion criteria: pre-eclampsia; ectopic pregnancy; preterm delivery; systemic rheumatic diseases. Detailed information was collected from each subject, and the inquiry included personal general condition, clinical symptoms, and past medical history and family medical history (Table 1). The study was approved by the Ethics Committee of Shanxi Medical University (ethical approval number: 2019LL152), and each participant signed an informed consent form before enrollment.

**Table 1:** Comparison of general information between the case and control groups.

Group	Total number of cases	Age (yr)	BMI (kg/m^2^)	Number of spontaneous abortions	Organic lesions of the uterus	Oral contraceptives (case)	Smoking	Drinking	Previous medical history
Case group	80	32.21 ± 4.24	20.53 ± 2.13	3.59 ± 1.35	None	2	None	None	None
Control group	90	31.18 ± 6.17	20.40 ± 2.16	0	None	1	None	None	None
*t*	–	1.253	0.394	25.237	–	–	–	–	–
*P*	–	.242	.593	<.001	–	–	–	–	–

*P* < .05 indicates that the difference is statistically significant. Age and BMI were not significantly different in the case and control groups. The number of spontaneous abortion was significantly different between the case and control groups.

## 3. Methods

### 3.1. Selection of loci

Peripheral blood from 10 pairs of URSA patients matched for gestational weeks (case group) and peripheral blood from normal pregnant women (control group) were used as study subjects. The above case and control groups were subjected to high-throughput sequencing, whole-exome gene detection, analysis of TGF-β signaling pathway-related genes and screening of polymorphic loci with frequency differences between the case and control groups, and verification of the polymorphic loci by Sanger sequencing.

Whole exon sequencing steps:

1. *Extraction of blood whole genomic DNA*: Blood samples (5 mL) were drawn and collected in EDTA-coated collection bottles (purple tubes), and later frozen in a −80°C refrigerator for DNA extraction in the laboratory within 1 to 2 weeks. Whole genomic DNA was extracted using a blood genomic DNA extraction kit (Tiangen, Beijing, China), following the instructions in the kit. QC: total genomic DNA > 10 ng; 260/280 between 1.8 and 2.0, 260/230 between 1.8 and 2.2; no degradation by electrophoresis detection.
2. *Whole-exome sequencing*: The exome sequences were captured by IDT’s xGen Exome Research Panel v1.0 capture probes, followed by high-throughput sequencing (PE150) of the captured sequences by NovaSeq 6000 series sequencers, requiring more than 99% sequencing coverage. The sequencing process was performed by Beijing Zhiying Oriental Translational Medicine Research Center.
3. *Analysis of high-throughput sequencing results*: The reference genome (GRCh37/hgl9) was compared with the results after high-throughput sequencing, and all variants were obtained after the comparison, and the polymorphic loci with frequency differences in the case and control groups were screened by screening all base variants (MAF value > 5%).

Sanger sequencing validation: polymorphic loci detected by whole exome sequencing were amplified after designing PCR primers using the Primer-BLAST tool on the NCBI website. The PCR reaction system was: 2 × PCR Mix 25 µL + ddH_2_O 20 µL + DNA 1 µL + 2 µL each of upstream/downstream primers, a total of 50 µL. IL-6 (-174G/C) reaction conditions were: 94°C for 3 minutes; 57°C for 30 seconds, 59°C for 30 seconds, 61°C for 1 minute, repeat 30 cycles; 72°C for 5 minutes; 4°C constant temperature. TGF-β1 (-509T/C) and SMAD1 (c.*149(exon7)A/C) reaction conditions were: 94°C for 3 minutes; 94°C for 30 seconds, 55°C for 30 seconds, 72°C for 1 minute, repeat 30 cycles; 72°C for 5 minutes; 4°C constant temperature. Finally, the PCR amplification products were purified, a DNA sequencing instrument (ABI, model 3730DX) was used for sequencing, and Comparison of the sequencing results with standard sequences by SeqMan software, then sequence mapping.

Primers were designed by applying the Primer-BLAST tool as follows:

Forward primers

**Table:** 

Forward primer ID	Forward primer sequence
IL-6(-174G/C)_W1_F	AGTTCTACAACAGCCGCTCA
TGF-β1(-509T/C)_W1_F	AAAGCGGGTGATCCAGATGC
SMAD1(c.*149(exon7)A/C)_W1_F	AGGGAGGAAAGATGCATAGCTT

Reverse primers

**Table:** 

Reverse primer ID	Reverse primer sequence
IL-6(-174G/C)_W1_R	AGAAGGAGTTCATAGCTGGGC
TGF-β1(-509T/C)_W1_R	GGATGGCACAGTGGTCAAGA
SMAD1(c.*149(exon7)A/C)_W1_R	GACAGCAAGTATGGTCAGCA

The polymorphic loci potentially associated with miscarriage were screened and selected for validation through Pubmed, clinvar, omim, China Knowledge Network, and Wanfang databases with the same inclusion and exclusion criteria as those in this paper.

### 3.2. Flight mass spectrometry experiments on the selected 7 gene loci

#### 3.2.1. Collection and processing of blood samples.

In addition to the 10 pairs of case and control groups for high-throughput sequencing, blood DNA was extracted from the remaining 70 case groups and 80 control groups.

#### 3.2.2. Whole genomic DNA extraction from peripheral blood.

Whole genomic DNA was extracted using the Blood Genomic DNA Extraction Kit (Tiangen), the same steps as for whole exome sequencing to extract blood whole genomic DNA.

#### 3.2.3. Amplification of target genes.

Primer design, synthesis: The amplification primer carries a 10 mer tag (ACGTTGGATG) at the 5’ end of the primer, which aims to make the first round of PCR primers ≥ 30bp (the range of mass spectrometry peak detection is between 4500 and 9000 Da, while the average molecular weight of bases is 300 Da, so the corresponding nucleotide detection range is 13 ~30 bp, so that the first round of PCR primers will not appear in the mass spectrogram even if they are not completely digested by the SAP that follows). The primer sequences are as follows:

Forward primers:

**Table:** 

Forward primer ID	Tag sequence	Forward primer sequence
TGF-β1(-509T/C)_W1_F	ACGTTGGATG	AAGAGGGTCTGTCAACATGG
TNFRSF4 (c.193C/T)_W1_F	ACGTTGGATG	TGACCACGTCGTTGTAGAAG
TGF-β1(c.133C/T)_W1_F	ACGTTGGATG	AGCTTGGACAGGATCTGGC
TNF-α (-308G/A)_W1_F	ACGTTGGATG	GCATCCTGTCTGGAAGTTAG
IL-6(-174G/C)_W1_F	ACGTTGGATG	AGTGGTTCTGCTTCTTAGCG
TGF-β1(c.328C/T)_W1_F	ACGTTGGATG	AGCTCACCGTTGTGGGTTTC
SMAD1(c.*149(exon7)A/C)_W1_F	ACGTTGGATG	TTGAGAACTGACAAAGGAGC

Reverse primers:

**Table:** 

Reverse primer ID	Tag sequence	Reverse primer sequence
TGF-β1(-509T/C)_W1_R	ACGTTGGATG	AGGAGAGCAATTCTTACAGG
TNFRSF4 (c.193C/T)_W1_R	ACGTTGGATG	TCTGCTGCAGGCAACGGGAT
TGF-β1(c.133C/T)_W1_R	ACGTTGGATG	ATCCACCTGCAAGACTATCG
TNF-α (-308G/A)_W1_R	ACGTTGGATG	CTGATTTGTGTGTAGGACCC
IL-6(-174G/C)_W1_R	ACGTTGGATG	GATTGTGCAATGTGACGTCC
TGF-β1(c.328C/T)_W1_R	ACGTTGGATG	TGTACAACAGCACCCGCGAC
SMAD1(c.*149(exon7)A/C)_W1_R	ACGTTGGATG	CAGTATGTTACCAAGGTGTG

PCR amplification, SAP purification, extension, or transcriptional cleavage; specific steps:

1. Add PCR Mix 3uL (30 µL, HS) → add DNA 2uL (20 µL, HS) (total volume 5 µL/50 µL) → instantaneous centrifugation, amplification [PCR (95°C 2 minutes-[95°C 30 seconds-56°C 30 seconds-72°C 60 seconds] × 45–72°C 5 minutes-forever)];
2. Take out amplification reagent, instantaneous centrifugation, add SAP Mix 2 µL (20 µL, HS) (total volume 7 µL/70 µL) → instantaneous centrifugation, amplification [PCR (37°C 40 minutes-85°C 5 minutes-4°C forever)];
3. Take out amplification reagent, instantaneous centrifugation, add extension Mix 2 µL per well (total volume 9 µL) → instantaneous centrifugation, amplification [PCR (94°C 30 seconds-[94°C 5 seconds-<52°C 5 seconds-80°C 5 seconds>×5] × 40–72°C 3 minutes-4°C forever)];
4. Take out the amplification reagents, instantaneous centrifugation, and overnight at 4°C.

#### 3.2.4. SNP locus flight mass spectrometry.

Application of Sequenom MassArray flight mass spectrometer to determine target gene SNP locus genotype: provide DNA detection locus information; sample integrity testing and pretreatment; point sample.

Specific steps:

1. Adding 41 μL of water to the wells with samples, sealing the membrane, and centrifuging → spreading clean resin on a 96/15 mg dimple plate → adding 15 mg of clean resin to the wells and sealing the membrane → shaking for 40 minutes on a rotary mixer → centrifuging at 4000 rpm for 5 minutes;
2. Importing assay → programming the board in Typer 4.0 software;
3. change the single needle on the spotter → add 50% anhydrous ethanol on the spotter → create a Mapping file on the spotter → create a Method file on the spotter → add 60 µL of standard (each time a new chip is used);
4. Put the chip into the spotter → put the 96-well plate or 8-tube into the spotter and spot the sample → put the chip into the analyzer → punch the mass spectrum → save the data.

### 3.3. Statistical analysis

All statistical analyses were performed using the Statistical Package for the Social Sciences (SPSS) version 26.0. Measurements in the general data of the study population (including confounding variables such as age and BMI) conformed to normal distribution and were expressed as mean ± SD, and comparisons were made using the independent samples *t* test. Differences in genotype frequencies were assessed using the chi-square test, whereas differences in allele frequencies were estimated using Fisher’s exact test with a 95% confidence interval. *P* value of less than .05 was considered statistically significant. Odds ratios were used to describe the strength of association between URSA and allele frequencies.

## 4. Results

**Figure 1.**
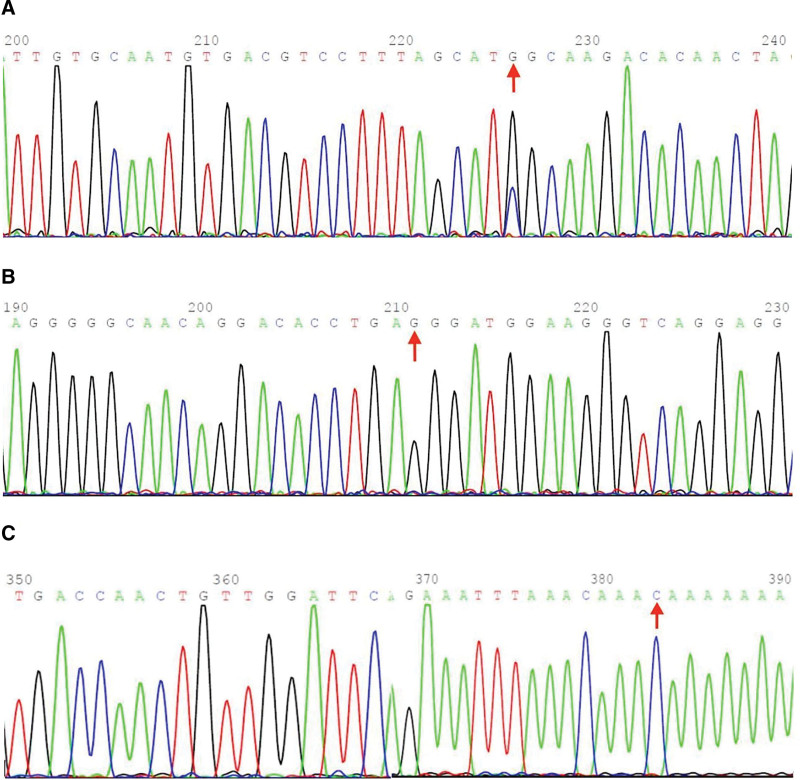
Sanger sequencing validation peak map of polymorphic loci screened by high-throughput sequencing. Note: High-throughput sequencing of 10 pairs of URSA women and normal control women screened 3 polymorphic loci that differed in frequency between case and control groups, namely IL-6 gene -174G/C locus (rs1800795), TGF-β1 gene -509T/C locus (rs1800469), and SMAD1 gene c.*149(exon7)A/C locus (rs28397904). Subsequently, Sanger sequencing was performed to verify the variant. (A) Sanger sequencing result of the IL-6 gene polymorphism locus: there is a IL-6 gene -174G/C heterozygous mutation; (B) Sanger sequencing result of the TGF-β1 gene polymorphism locus: there is a TGF-β1 gene -509T/C homozygous mutation; and (C) Sanger sequencing result of the SMAD1 gene polymorphism locus: there is a homozygous mutation of c.*149(exon7)A/C in the SMAD1 gene.

1. By analyzing the data from high-throughput sequencing and whole exon detection of 10 pairs of case and control groups, 3 polymorphic loci with differences in frequency between the case and control groups were screened out, namely IL-6 gene -174G/C locus (rs1800795); TGF-β1 gene -509T/C locus (rs1800469); SMAD1 gene c.*149(exon7)A/C locus (rs28397904) (Table 2), followed by Sanger sequencing proved this variant results (Fig. 1).
2. Four polymorphic loci that may be associated with miscarriage were screened by Pubmed, clinvar, omim, China Knowledge Network, and Wanfang databases, namely TNF-α gene -308G/A locus (rs1800629); TGF-β1 gene c.328C/T locus (rs1555755242); TGF-β1 gene c.133C/T locus (rs1555755308); TNFRSF4 gene c.193C/T locus (rs587777075). Reports of the association of these loci with miscarriage exist in the database, and these loci were selected for validation.
3. Flight mass spectrometry experiments and gene polymorphism analysis were performed for the selected 7 gene loci in the remaining 70 URSA patients and 80 normal controls except for high-throughput sequencing. By calculating the genotype and allele frequencies and their corresponding P values (Table 3), the results showed that the IL-6 gene -174G/C locus (rs1800795, located on exon 2, reference sequence NM_000600.5, chromosome position chr7:22727026 GRCh38.p14) and the TGF-β1 gene -509T/C locus (rs1800469, located on exon 1, reference sequence NM_000660.7, chromosome position chr19:41354391 GRCh38.p14) had significant differences in genotype and allele distribution frequencies between the case and control groups (*P* < .05). The risk of disease was 2.636 and 3.231 times higher in individuals carrying the C allele and CC genotype of the IL-6 gene -174G/C locus than in those carrying the G allele and GG genotype, respectively. The risk of disease in individuals carrying the T allele and TT genotype at the TGF-β1 gene -509T/C locus was 1.959 and 3.609 times higher than that of individuals carrying the C allele and CC genotype, respectively. Therefore, the IL-6 gene -174G/C locus (rs1800795) and TGF-β1 gene -509T/C locus (rs1800469) polymorphisms were considered to be possibly associated with URSA. TNF-α gene -308G/A locus (rs1800629), TGF-β1 gene c.328C/T locus (rs1555755242), TGF-β1 gene c.133C/T locus (rs1555755308), SMAD1 gene c.*149(exon7)A/C locus (rs28397904), and TNFRSF4 gene c.193C/T locus (rs587777075) polymorphisms were not significantly different between the case and control groups (*P* > .05), and it is speculated that their gene polymorphisms may not be related to URSA.

**Table 2:** High-throughput sequencing results of 10 pairs of case and control groups.

Genes	Case group (cases)	Control group (cases)	Type of variation
IL-6 gene -174G/C locus (rs1800795)	2	1	Heterozygote
TGF-β1 gene-509T/C locus (rs1800469)	0	2	Pure zygotes
SMAD1 gene c.*149(exon7)A/C locus (rs28397904)	1	2	Pure zygotes

Polymorphisms of gene loci in Table 2 differed in the case and control groups. IL-6 gene -174G/C locus is a heterozygous mutation, and TGF-β1 gene -509T/C locus and SMAD1 gene c.*149(exon7)A/C locus are homozygous mutations.

**Table 3:** Allele and genotype frequency distribution of polymorphic loci of TGF-β signaling pathway-related genes.

SNP name			URSA group (cases, %)	Control group (cases, %)	OR value	95% CI	χ^2^ value	*P* value
SMAD1 gene								
c.*149(exon7)A/C	Allele	A	110 (78.6)	125 (78.1)	1.0			
rs28397904		C	30 (21.4)	35 (21.9)	0.974	0.561–1.690	0.009	.925
	Genotype	AA	49 (70.0)	57 (71.25)	1.0			
		AC	12 (17.1)	11 (13.75)	1.269	0.514–3.130	0.268	.605
		CC	9 (12.9)	12 (15.0)	0.872	0.339–2.244	0.08	.777
IL-6 gene								
-174G/C	Allele	G	110 (78.6)	145 (90.6)	1.0			
rs1800795		C	30 (21.4)	15 (9.4)	2.636	1.352–5.139	8.508	.004
	Genotype	GG	52 (74.3)	70 (87.5)	1.0			
		CG	6 (8.6)	5 (6.25)	1.615	0.468–5.581	0.583	.445
		CC	12 (17.1)	5 (6.25)	3.231	1.072–9.737	4.697	.03
TNF-α gene								
-308G/A	Allele	G	119 (85.0)	133 (83.1)	1.0			
rs1800629		A	21 (15.0)	27 (16.9)	0.869	0.467–1.618	0.195	.659
	Genotype	GG	55 (78.6)	63 (78.75)	1.0			
		AG	9 (12.8)	7 (8.75)	1.473	0.514–4.216	0.525	.469
		AA	6 (8.6)	10 (12.5)	0.687	0.235–2.013	0.472	.492
TNFRSF4 gene								
c.193C/T	Allele	C	140 (100)	160 (100)				
rs587777075	Genotype	CC	70 (100)	80 (100)				
TGF-β1 gene								
-509T/C	Allele	C	45 (32.1)	77 (48.1)	1.0			
rs1800469		T	95 (67.9)	83 (51.9)	1.959	1.223–3.137	7.904	.005
	Genotype	CC	8 (11.4)	21 (26.25)	1.0			
		CT	29 (41.4)	35 (43.75)	2.175	0.840–5.632	2.618	.106
		TT	33 (47.2)	24 (30.0)	3.609	1.369–9.515	7.078	.008
TGF-β1 gene								
c.328C/T	Allele	C	140 (100.0)	160 (100.0)				
rs1555755242	Genotype	CC	70 (100.0)	80 (100.0)				
TGF-β1 gene								
c.133C/T	Allele	C	140 (100.0)	160 (100.0)				
rs1555755308	Genotype	CC	70 (100.0)	80 (100.0)				

*P* < .05 indicates that the difference is statistically significant. The -174G/C locus of the IL-6 gene and the -509T/C locus of the TGF-β1 gene showed significant differences in genotype and allele frequencies between the case and control groups. The genotype and allele frequencies of the other genes have no significant difference between the case and control groups.

## 5. Discussion

Pregnancy is a complex process involving many signaling pathway molecules, and the involvement of TGF-β signaling pathway-related genes in spontaneous abortion has been reported.^[14]^ TGF-β family factors are extensively involved in embryogenesis, not only in regulating cell apposition, proliferation and migration but also in a series of processes such as cell differentiation and death.^[5,7,16]^ There is growing evidence that the TGF-β family plays an important role in embryogenesis, tissue homeostasis and the pathogenesis of many diseases.^[17]^

Gene polymorphism analysis is an important tool in genomic research, mainly to detect relevant gene polymorphisms to understand the mechanism of disease, to speculate the degree of association between the studied genetic marker and a genetic disease susceptibility locus, and to play an important role in disease diagnosis, individualized treatment, and disease assessment. Several studies have shown a possible association between polymorphisms in TGF-β1, TGF-β2, and TGF-β signaling pathway genes and susceptibility to many diseases.^[18–21]^ Many genetic studies on TGF-β signaling pathway gene polymorphisms also suggest their possible involvement in the pathogenesis of URSA,^[22–24]^ however, these reports are controversial and inconclusive. In the present study, we screened 7 related gene loci on the TGF-β signaling pathway for study, and the results showed that the IL-6 gene -174G/C locus (rs1800795) and the TGF-β1 gene -509T/C locus (rs1800469) had significant differences in genotype and allele distribution frequencies between the case and control groups, and presumably their genetic polymorphisms may be associated with URSA. The differences in genotype and allele distribution frequencies of the remaining 5 gene loci were not statistically significant.

### 5.1. IL-6 gene -174G/C (rs1800795)

Cytokine-mediated immune mechanisms play an important role in normal pregnancy, and either the site of cytokine expression or the amount of protein may lead to trophoblast-endometrial interactions and eventual progression to URSA.^[25–27]^ Although the relevance of polymorphisms in genes encoding cytokines such as IL-6 to URSA has been studied, their role and relevance remain unclear.^[26,28,29]^ Von Linsingen conducted a study on IL-6 gene -174G/C locus polymorphism and URSA in southern Brazil and confirmed significant differences in IL-6 gene expression between women with URSA and control women in southern Brazil and that IL-6 levels were lower in women with URSA.^[30]^ Demirturk F showed that IL-6 gene -174G/C polymorphism was associated with an increased risk of URSA in Turkish women.^[23]^ However, Silvia Daher in the study there was no evidence that IL-6 gene -174 locus polymorphism was associated with URSA in Brazilian Caucasian women.^[31]^

The IL-6 gene is located in chromosome region 7p21 and is an important factor in the TGF-β signaling pathway.^[32]^ IL-6 is widely found in the female reproductive tract and gestational tissues and has a very important role in embryo implantation and placental development.^[33,34]^ The gene has highly complex transcriptional regulation, and a single nucleotide change from G to C may lead to alterations in the transcription of the gene, as this nucleotide change creates a potential binding site for the transcription factor NF-1, which is a blocker of gene expression in HeLa cells.^[35]^ Meanwhile, common SNPs in promoters that affect IL-6 expression are the -174 and -634 polymorphic loci.^[28,35]^ A common C/G polymorphism at the 174 bp nucleotide position upstream of the transcription start site within the IL-6 gene promoter has been reported to affect IL-6 expression and may lead to abortion.^[33,36]^ Our research centers on the association between polymorphisms in the promoter region of the IL-6 gene -174G/C locus and the occurrence of URSA. In addition, polymorphism studies of the IL-6 gene showed that polymorphisms at the -572G/C, -597G/A, -1363G/T, and -2954G/C loci have a synergistic effect on IL-6 gene transcription^[23]^ and that IL-6 genotypes may be associated with the development of juvenile systemic arthritis, Kaposi’s sarcoma, and atherosclerosis.^[37–39]^

The results of our study showed that the frequency of genotypes and alleles at the -174G/C locus of the IL-6 gene was significantly different in patients with URSA compared with controls (*P* < .05), and the frequency of the -174 locus CC genotype and allele C of the IL-6 gene was statistically higher in patients with URSA, suggesting that the pure zygote CC and allele C may be the pathogenic factors of URSA, with previously published data having the same results.^[23,27,40]^ Many scholars have suggested that the IL-6 gene SNP-174 locus containing the C allele has lower IL-6 protein and mRNA expression in serum than individuals purely containing the G allele,^[33,41]^ and the plasma concentration of IL-6 in women with URSA is lower than that in women with successful pregnancy.^[23]^ So it is assumed that the C allele of the IL-6 gene-174 gene locus is a pathogenic factor for URSA. The inconsistency between IL-6 gene polymorphisms and protein level expression may be due to the influence of other IL-6 gene polymorphisms or IL-6 protein regulatory mechanisms.^[42]^

### 5.2. TNF-α gene -308G/A (rs1800629), TNFRSF4 gene c.193C/T (rs587777075)

Tumor necrosis factor-α (TNF-α) is a pro-inflammatory cytokine produced by different immune cells, including antigen-stimulated T cells, lymphocytes, and NK cells. Certain loci polymorphisms of TNF are associated with TNF secretion and pregnancy complications,^[27,29]^ including URSA. Manzoor assessed the risk of genetic variation in the TNF-α gene -308G/A locus in patients with RSA in the Kashmiri (North India) population, and found that the genotype AA was significantly associated with RSA cases. The frequency of allele A in the case group was also higher, indicating that the TNF-α gene -308G/A mutation was significantly associated with the susceptibility to pregnancy loss.^[43]^ In Iranian Azeri Turkish women, the -857C/C variant of the TNF-α gene may represent a protective role for RSA and the -857C/T variant may be a genetic risk factor for the development of RSA.^[44]^

The TNFRSF4 gene encoding the TNF receptor superfamily may be a risk factor for URSA.^[45,46]^ The protein encoded by the TNFRSF4 gene is a member of the TNF receptor superfamily, a member of the TNF receptor superfamily called GITR. It has been shown that GITR interacts with the pro-apoptotic protein Siva, leading to cell death.^[46]^ Mouse gene knockout studies have shown that the TNF receptor superfamily plays a role in CD4^+^ T cell response and T cell-dependent B cell proliferation and differentiation.^[47]^ Byun identified the c.193C/T mutation in the TNFRSF4 gene, leading to arg65-to-cys substitution, which is associated with immunodeficiency.^[48]^ Clinvar suggested that the TNFRSF4 gene c.193C/T locus was the pathogenic locus of abortion. However, our results showed that the TNF-α gene -308G/A locus, TNFRSF4 gene c.193C/T locus genotype and allele distribution frequency were not statistically significant (*P* > .05) when comparing the case and control groups, and failed to demonstrate that their genetic polymorphisms were associated with URSA.

### 5.3. TGF-β1 gene -509T/C (rs1800469), c.328C/T (rs1555755242), c.133C/T (rs1555755308)

TGF-β includes 3 subtypes: TGF-β1, TGF-β2 and TGF-β3. It is a pleiotropic cytokine that plays a role in tissue fibrosis, wound healing and embryonic development.^[49,50]^ TGF-β1 gene is located on chromosome 19q13. It is a cytokine with a variety of biological functions and has been widely studied.^[51]^ TGF-β1 plays an important role in regulating immune cell differentiation, maintaining immune cell function and homeostasis.^[52]^ The metaphase immune microenvironment determines the maintenance of normal pregnancy, in which the dynamic balance between Th1 and Th2 cytokines plays a key role.^[53]^ In normal pregnancy, Th2 is dominant and Th1 is inhibited. When Th1 cytokines are overexpressed, it will lead to abortion.^[54]^ TGF-β1 is a Th2-type cytokine that regulates uterine blood flow and the expression of vascular endothelial growth factor, promotes endometrial tolerance and contributes to embryo implantation, thereby affecting the outcome of pregnancy.^[55]^ Moreover, single nucleotide polymorphism of the TGF-β1 gene is associated with polycystic ovary syndrome in Chinese women.^[27]^ Increased levels of TGF-β1 and level reduction of TGF-β1 receptors may lead to polycystic ovary syndrome and ovarian hyperstimulation.^[56,57]^

The relationship between TGF-β1 gene polymorphisms and URSA has been reported less frequently and with variable results. Amani found that TGF-β1 gene polymorphism at the -509C/T locus was not associated with URSA in a study of 111 women with URSA in southern Iran.^[58]^ Afrah showed that the results of an experiment in Saudi women with TGF-β1 gene -509T/C locus allele and genotype frequencies were not significantly different between women with URSA and healthy controls.^[27]^ Li Xia demonstrated that the TGF-β1 gene -509C/T polymorphic locus is associated with URSA and its TT genotype may be a susceptibility factor for URSA in Han Chinese women in Ningxia.^[59]^ Huang found an increased risk of URSA for the CT genotype of the -509C/T locus of the TGF-β1 gene for the Chinese Guangdong population, while the TT genotype may be a protective factor for RSA, which is different from the results of Li Xia’s study.^[60]^ Our study showed that the distribution frequency of allele or genotype of TGF-β1 gene -509T/C locus was significantly different between the case group and the control group (*P* < .05). TT genotype and allele T had a higher frequency in URSA, which may be the pathogenic factor of URSA.

Kotlarz D found that the TGF-β1 gene (c.328C/T, p.Arg110Cys) and (c.133C/T, p.Arg45Cys) is located in the sequence encoding the LAP structural domain.^[61]^ Both loci are associated with immune dysregulation, which may lead to diseases such as arthritis, encephalopathy, inflammatory bowel disease, recurrent infections, and immunodeficiency. In the Clinvar database for the miscarriage causative locus and the miscarriage probable causative locus, respectively. However, the results of this experiment showed no statistically significant difference in allele frequency between the TGF-β1 gene c.328C/T locus and the TGF-β1 gene c.133C/T locus in the case and control groups for comparison.

### 5.4. SMAD1 gene c.*149(exon7)A/C (rs28397904)

There are 2 pathways used by TGF-β family members for signal transduction, one is SMADs-dependent and the other is a SMADs-independent pathway^[5,62]^ that both coordinate with each other and function together to regulate signal transduction.^[63]^ For SMAD-dependent signaling, SMAD proteins are the main mediators of TGF-β signaling.^[64]^ The 8 SMADs identified in vertebrates consist of 5 R-SMADs, of which SMAD1/5/8 are responsive to bone morphogenetic protein and growth differentiation factor,^[65]^ of which SMAD2/3 is activated by TGF-β and activin stimulation.^[66]^

SMAD2/3 is a downstream protein of the TGF-β signaling pathway, which transports signals from the cell membrane to the nucleus, binds DNA, and controls the expression of target genes.^[67]^ In an experimental model of Toxoplasma excretory-secretory antigen induced miscarriage in mice, it was found that excretory-secretory antigen likely inhibited the phosphorylation of SMAD2/3/4 and negatively regulated the expression of Foxp3 to suppress Treg function, ultimately leading to miscarriage.^[68]^ SMAD3 is associated with intrauterine adhesions, which can also lead to infertility and URSA.^[69]^ A study conducted by Zhao showed that reduced levels of SMAD3 expression in mice may lead to URSA.^[70]^ Bremm conducted a case-control study of 149 women with URSA and 159 control women and found that the C allele of the C.207-19370T > C (rs17293443) locus of the Smad3 gene was less frequent in the case group than in the control group and that CC and CT genotypes were associated with a lower risk of URSA, demonstrating that Smad3 gene polymorphisms may influence URSA susceptibility.^[24]^

SMAD1/5 induces bone repair and bone and cartilage formation, and it is essential in embryonic development, mesoderm formation and the developmental shaping of various other organ systems. SMAD1/5 gene research has focused on the regulatory effects on the skeletal system, nervous system, and tumors, but little research has been done on the relationship with recurrent miscarriage. Because high-throughput sequencing showed that there was a frequency difference between cases and controls at the c.*149(exon7) A/C locus of the SMAD1 gene, we focused on the relationship between the polymorphism at the c.*149(exon7) A/C locus of the SMAD1 gene and recurrent miscarriage in this experiment. The results showed that there was no statistically significant difference in SMAD1 gene c.*149(exon7) A/C locus polymorphism between the case and control groups. Although there was a frequency difference in SMAD1 gene c.*149(exon7) A/C locus between the case and control groups during high-throughput sequencing, the difference was not significant after expanding the sample size for flight mass spectrometry experiments.

### 5.5. Genotyping method

As an efficient genotyping method, high-throughput sequencing technology has the characteristics of high throughput, fast and accurate. However, the disadvantages are high cost and complex data processing, which are generally used for genetic screening. The flight mass spectrometry experiments for genotyping are separated by the difference in migration rate of ions in the electric field, and the separated ions enter the mass spectrometry region for mass analysis to determine their genotypes. The method is characterized by small sample size, short time-consuming, good reproducibility, and low cost. It is suitable for the clinical diagnosis and verification of disease-related biomarkers in the population. Therefore, the combined method of the 2 technologies is innovative and feasible. Although techniques such as Sanger sequencing or restriction fragment length polymorphism can also detect gene locus polymorphism, Sanger sequencing is slow, expensive, and requires a lot of manual operation. restriction fragment length polymorphism has certain limitations in DNA sequence analysis and identification, which is dependent on the enzymatic cleavage site of restriction enzymes, limited by the length of the enzymatic fragment, and unable to determine the exact location of the enzymatic cleavage site.

### 5.6. Limitations of the study

The study of the relationship between gene polymorphisms and URSA, in addition to the influence of other gene loci and protein regulation, may also be related to the selection of the study population, the size of the sample size, as well as the geographic location and race. In this paper, the study population was selected from the Han Chinese population in North China, and the sample size was small, with racial and regional homogeneity. In future studies, we need to expand the sample size, conduct external validation in different time, region and race populations, and also consider the interactions with other cytokine gene polymorphisms and possible genetic chain imbalance of genes to eliminate the limitations of the study as much as possible.

## 6. Conclusion

Our results indicate that polymorphisms at the -174G/C locus of the IL-6 gene and the -509T/C locus of the TGF-β1 gene are associated with the risk of URSA in local patients from Shanxi, China, revealing that polymorphisms in the TGF-β signaling pathway genes have a correlation with URSA. Moreover, the previous study of our group showed that the mRNA expression of IL-6 and TGF-β1 genes decreased more than 2-fold in URSA embryonic tissues compared with the control group, suggesting that there is a significant difference between the expression of the above genes in the TGF-β signaling pathway in URSA embryonic tissues and normal embryonic tissues. Studies have shown that the TGF-β signaling pathway can regulate the expression of cytokines, and the role of cytokines in URSA is more clear. Studying the interaction between the TGF-β signaling pathway and cytokines can provide a better understanding of the mechanism of URSA, provide new therapeutic targets for drug development, improve the treatment of URSA, and improve the fertility of URSA patients. In the future research, we will study the transcription level and protein expression of these polymorphic gene loci through relevant experiments, and explore the association between the above gene polymorphisms and immunity, so as to provide a new basis for the diagnosis and treatment of URSA.

## Acknowledgments

We are very grateful to our patients for their cooperation.

## Author contributions

**Conceptualization:** Huiqin Xue, Xinyan Li, Yuping Zhang.

**Data curation:** Qiaoyin Tang.

**Formal analysis:** Jinsong Jiang, Jingbo Gao, Xinyan Li.

**Funding acquisition:** Huiqin Xue, Hongyong Lu.

**Investigation:** Min Guo, Hongyong Lu, Jianrui Wu.

**Methodology:** Huiqin Xue, Hongyong Lu.

**Supervision:** Huiqin Xue, Xiayu Sun, Yuping Zhang.

**Validation:** Jinsong Jiang, Min Guo, Qiaoyin Tang, Jianrui Wu.

**Writing – original draft:** Jinsong Jiang.

**Writing – review & editing:** Jinsong Jiang, Jingbo Gao, Xiayu Sun.
